# Alternative ankle–brachial assessments show no significant added value in predicting mortality of hypertensive patients

**DOI:** 10.1097/HJH.0000000000004255

**Published:** 2026-02-09

**Authors:** Endre Kolossváry, Tamás Ferenci, Zoltán Járai, Katalin Farkas

**Affiliations:** a Department of Angiology, St. Imre University Teaching Hospital; b Section of Angiology, Heart and Vascular Center, Semmelweis University; c Physiological Controls Group, John von Neumann Faculty of Informatics, Óbuda University; d Department of Statistics, Corvinus University of Budapest; e Department of Cardiology, St. Imre University Teaching Hospital, Budapest, Hungary

**Keywords:** ankle–brachial index, hypertension, mortality prediction, peripheral artery disease

## Abstract

**Background:**

Peripheral artery disease (PAD), assessed via the ankle–brachial index (ABI), is a recognized form of hypertension-mediated organ damage (HMOD). While alternative ABI calculations have shown improved sensitivity for PAD detection, their prognostic utility in hypertensive populations remains unclear.

**Methods:**

In this prospective cohort study of 21 875 hypertensive individuals (ÉRV Study), we compared the prognostic performance of three ABI-based approaches: standard ABI using the higher ankle pressure (ABI-HIGH), ABI using the lower ankle pressure (ABI-LOW), and multivessel ABI scoring (number of vessels with ABI ≤0.90). The primary endpoint was all-cause mortality, assessed over a median follow-up of 5 years using interval-censored Cox regression.

**Results:**

PAD prevalence was 14.4% using ABI-HIGH and 28.3% using ABI-LOW, with 13.9% of patients identified only by the latter. All PAD definitions were independently associated with mortality. ABI-LOW as a continuous variable demonstrated the strongest association (hazard ratio 1.87; 95% CI, 1.63–2.16). Multivessel ABI showed a dose–response relationship with mortality. However, overall discrimination was modest: time-dependent AUCs ranged from 0.608 to 0.635 for ABI-based models alone. When added to clinical predictors, ABI metrics improved the AUC to a range from 0.763 to 0.780, with added predictive value between 6 and 11%.

**Conclusion:**

In hypertensive individuals, ABI-LOW and multivessel scoring identify more PAD cases and are independently associated with mortality. However, their incremental value in mortality risk prediction is limited. Alternative ABI methods may assist in identifying higher risk subgroups warranting further vascular assessment.

## INTRODUCTION

Lower extremity peripheral artery disease (PAD) is recognized in current hypertension guidelines as a form of hypertension-mediated organ damage (HMOD), with potential implications for both clinical management and cardiovascular risk stratification [1,2]. While prior studies in the general population [3–5], patients with diabetes [6], and older adults [7] have demonstrated the prognostic relevance of PAD for all-cause mortality, coronary events, and cardiovascular mortality, data specific to hypertensive populations remain limited.

In a large hypertensive cohort followed over 3 years, individuals with an ankle–brachial index (ABI) of 0.41–0.90 had a significantly elevated mortality risk [relative risk, 1.53; 95% confidence interval (CI), 1.2–1.96], which was further increased in those with an ABI less than 0.40 (relative risk, 3.11; 95% CI, 1.94–4.98) [8,9]. Another study in an urban hypertensive population found that PAD and reduced cardiovascular health were independently associated with adverse outcomes, including increased risk of death and/or hospitalization (hazard ratio, 4.13; 95% CI, 1.26–13.47). [10] In our prior investigation (ÉRV Study), involving nearly 22 000 hypertensive individuals, PAD prevalence was 14.4% and was strongly associated with established atherosclerotic disease, impaired renal function, diabetes mellitus, hyperlipidemia, and smoking [11]. Over a 5-year follow-up, PAD independently predicted all-cause mortality (hazard ratio, 1.87; 95% CI, 1.63–2.16), with mortality risk increasing continuously and smoothly across ABI values [12].

All referenced studies utilized the standard ABI measurement with continuous-wave Doppler, calculated as the higher systolic ankle pressure (dorsalis pedis or posterior tibial artery) divided by the higher brachial pressure (ABI-HIGH). An ABI-HIGH 0.90 or less is widely accepted as the diagnostic threshold for PAD [13].

While the specificity of this method for detecting at least 50% arterial stenosis is high (83–99%) (reference tests: digital subtraction angiography, DSA, computed tomography angiography, magnetic resonance, angiography, or color duplex ultrasound), sensitivity is modest (20–79%), particularly among older adults, individuals with diabetes, or those with distal disease [14,15]. This problem is not only associated with inaccuracy in diagnosis but also affects cardiovascular risk prediction by potentially ignoring patients with substantial risk for cardiovascular events.

Alternative ABI calculations have been proposed to improve diagnostic sensitivity. One such approach involves using the lower ankle systolic pressure (ABI-LOW), which has been shown to detect additional cases of PAD and to identify individuals at increased risk for cardiovascular events [16,17].

ABI-LOW not only identifies more patients with PAD – who might otherwise be overlooked for preventive management – but may also serve as a better predictor of patients at higher risk for subsequent cardiovascular events compared to the reference population. Evidence from population-based cohorts [18], individuals without clinically overt cardiovascular diseases (CVDs) [19,20], and patients with advanced atherosclerosis who underwent an urgent or elective coronary angiogram for suspicion of ischemic heart disease [16,21] supports the utility of ABI-LOW in risk stratification in this context. However, in many of these studies, the predictive performance of ABI-HIGH and ABI-LOW was not significantly different.

A more detailed approach involves multivessel ABI (vessel-specific ABIs) assessment, which measures pressures in both ankle arteries and scores the number of affected vessels (from 0 to 4). This method has demonstrated a dose–response relationship with mortality, incident cardiovascular events, and cardiovascular mortality in primary prevention populations [20].

To date, neither ABI-LOW nor multivessel ABI approaches have been systematically evaluated in hypertensive populations. To our knowledge, the present study is the first to investigate whether alternative ABI assessment methods improve mortality risk prediction in individuals with hypertension.

## METHODS

### Study design

The Evaluation of Ankle/Brachial Index in Hungarian Hypertensives (ÉRV) program was a large-scale, multicenter, observational study with both cross-sectional and longitudinal components. The study was conducted between April 2007 and September 2014 in 55 hypertension outpatient clinics in Hungary. The study protocol was developed by the study coordinators and has been approved by the Central Ethical Review Board (Scientific Research Ethics Committee of the Medical Research Council of Hungary, chairperson: Zoltán Papp, protocol number: 22–35/2007–1018EKU, date: 29 February 2008). All procedures adhered to the Declaration of Helsinki. Detailed aspects of the study design have been published previously [11,12]. The present analysis utilized data from both baseline and follow-up assessments of the ÉRV cohort.

### Study population

During the initial enrollment phase, all consecutive patients aged 50–75 years with a diagnosis of hypertension who presented at participating centers were screened for inclusion. Recruitment was limited to a maximum of 40 patients per month per site. Follow-up frequency was determined by baseline ABI results: participants with normal ABI were re-evaluated at 5 years, whereas those with abnormal ABI values were assessed annually. By September 2008, a total of 21 892 individuals were invited to participate. Patients with advanced PAD (Fontaine stage III or IV) were excluded. Written informed consent was obtained from all participants.

### Risk factor assessment

At baseline and follow-up visits, a standardized medical history was obtained and physical examination, laboratory evaluation, and ABI measurement were performed. The following cardiovascular risk factors were recorded: smoking status, diabetes mellitus, hypercholesterolemia, waist circumference, family history of vascular disease, history of myocardial infarction, and history of ischemic stroke [22].

### Laboratory analysis

Venous blood samples were collected following an overnight fast for measurement of serum glucose, total cholesterol, triglycerides, uric acid, and creatinine. Measurement of HDL and LDL cholesterol and microalbuminuria was optional and performed at the discretion of the investigator.

### Ankle–brachial index measurement

All participating physicians and research staff were trained in standardized ABI measurement protocols, consistent with current guideline recommendations [13]. After a 5 min rest period, SBP was measured in both brachial arteries, followed by measurement of systolic pressures in the dorsalis pedis and posterior tibial arteries at the malleolar level bilaterally. A continuous-wave Doppler device (ELITE 200 Doppler, 5 MHz) was used uniformly across study sites. ABI assessors were blinded to participants’ clinical histories.

### Ankle–brachial index calculation

Three different ABI-based metrics were calculated in this study. The standard ABI (ABI-HIGH) was calculated by dividing the higher ankle systolic pressure (dorsalis pedis or posterior tibial artery) by the higher brachial systolic pressure [13]. Additionally, an alternative ABI (ABI-LOW) was calculated using the lower ankle systolic pressure (dorsalis pedis or posterior tibial artery) with the same brachial reference pressure. This methodology was adapted from Nead *et al.* [21].

For both ABI-HIGH and ABI-LOW, the lower of the two limb values was used in the analysis to represent the participant's ABI. PAD was defined as ABI 0.90 or less, and presence of PAD was coded as a binary variable based on either ABI-HIGH or ABI-LOW.

A third metric – multivessel ABI assessment – was performed following the approach described by Unkart *et al.* [20]. ABI was calculated separately for each of the four ankle arteries (bilateral dorsalis pedis and posterior tibial arteries), using the higher of the two brachial pressures as the denominator. Each vessel with an ABI 0.90 or less was considered to indicate PAD. The number of affected vessels (range: 0–4) was recorded and analyzed as a categorical variable.

**FIGURE 1:**
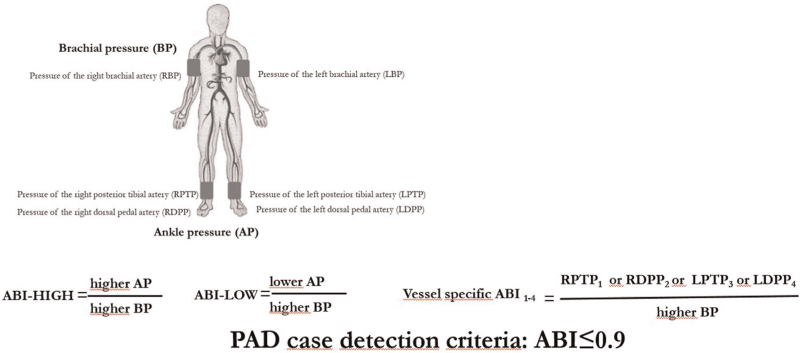
Different approaches in ankle–brachial index calculation [traditional ABI (ABI-HIGH), alternative ABI-ABI-LOW, vessel-specific ABIs). ABI, ankle–brachial index.

Thus, five ABI-derived variables were used in subsequent analyses: binary presence of PAD by ABI-HIGH, binary presence of PAD by ABI-LOW, continuous ABI-HIGH, continuous ABI-LOW, and categorical multivessel PAD score (0–4 vessels affected). The calculation methods are illustrated in Fig. 1.

### Study endpoint

The primary mortality endpoint was all-cause mortality. Cardiovascular mortality could not be determined because cause-of-death data were not available/reliably adjudicated in the source datasets; therefore, cause-specific mortality analyses were not performed.

### Statistical analysis

Categorical variables are presented as frequency (percentage), and continuous variables are presented as mean ± standard deviation (SD).

Due to the unavailability of exact dates of death, an interval-censored survival model was applied [23]. For individuals alive at the last follow-up visit, censoring was assumed at the date of that visit. For individuals known to be deceased, survival time was assumed to lie between the dates of the last recorded visit and the date of the next scheduled visits (interval censoring). Survival data were modeled using a semi-parametric Cox proportional hazards model adapted for interval-censored data [24]. To estimate confidence intervals (CIs), 1000 bootstrap samples were generated for each model.

Continuous covariates were modeled using natural cubic splines with four degrees of freedom to allow for potential nonlinear relationships with the outcome. Multiple multivariable Cox models were constructed, differing in how PAD was defined and how ABI was incorporated.

Models either contained only the PAD-related variable as predictor or were adjusted for baseline covariates. In the latter case, covariates including age, sex, BMI, SBP, DBP, and medical history of diabetes, myocardial infarction, stroke, and renal dysfunction. For comparability, all models were estimated on the same dataset, that is, the one where cases with missing variables in the clinical covariates were omitted. Models were specified as follows:

1. Model 1: PAD defined as a binary variable based on ABI-HIGH ≤ 0.90 (standard approach).
2. Model 2: PAD defined as a binary variable based on ABI-LOW ≤ 0.90 (alternative approach).
3. Model 3: ABI-HIGH as a continuous variable.
4. Model 4: ABI-LOW as a continuous variable.
5. Model 5: Multivessel PAD score (0–4 vessels with ABI ≤ 0.90), treated as a categorical variable.

Hazard ratios with 95% CIs are reported for categorical variables. For continuous predictors, estimated log-hazard curves with 95% CIs were plotted. In these plots, each predictor was varied across its empirical range (1st to 99th percentile), while other variables were fixed at their median (for continuous variables) or mode (for categorical variables). *P* values were derived using the likelihood ratio test.

Time-dependent receiver operating characteristic (ROC) curves and corresponding areas under the curve (AUCs) were computed using the method of Beyene and El Ghouch [25].

To quantify the added predictive value of PAD-related metrics beyond conventional clinical risk factors, likelihood ratio *χ*^2^ statistics were compared between nested models. The ratio of the *χ*^2^ statistic of the full model (including the PAD index) to the reduced model (without the PAD index) was used to calculate model adequacy. The fraction of new information was computed as 1 minus the adequacy, following the approach of Harrell and coworkers [26]. This allowed quantification of the unique contribution of ABI metrics in enhancing mortality risk prediction.

All analyses were conducted using R version 4.5.1 (R Foundation for Statistical Computing) with the icenReg package (version 2.0.16) [27]. The complete analysis code is publicly available at: https://github.com/ferenci-tamas/ERVstudy.

## RESULTS

A total of 21 892 hypertensive patients were initially enrolled in the ÉRV study. After excluding 17 patients due to missing outcome or ABI data, 21 875 individuals comprised the final analytic cohort.

Using the traditional ABI calculation based on the higher ankle pressure (ABI-HIGH), PAD was identified in 14.4% of participants (*n* = 3150). Within this group, the distribution of affected vessels (dorsal pedal and posterior tibial arteries bilaterally) was as follows: one-vessel in 0.0%, two-vessel in 26.8%, three-vessel in 26.4%, and four-vessel involvement in 46.8%.

When the alternative ABI calculation based on the lower ankle pressure (ABI-LOW) was applied, PAD prevalence increased to 28.3% (*n* = 6198). Notably, 13.9% of patients (*n* = 3048) were identified exclusively by the ABI-LOW method. Among those with ABI-LOW 0.9 or less, the distribution of vessel involvement was 36.3% with one-vessel, 27% with two-vessel, 13.2% with three-vessel, and 23.5% with four-vessel manifestations.

The distributions of ABI values for both traditional and alternative methods are illustrated in Supplementary Figure 1, and the relationship between ABI-HIGH and ABI-LOW values is depicted in Supplementary Figure 2.

Baseline characteristics of the study population are presented in Table 1, stratified into three groups: patients without PAD, patients with PAD identified by the traditional ABI-HIGH ≤ 0.9 criterion, and patients with PAD detected exclusively by the alternative ABI-LOW ≤ 0.9 criterion.

**TABLE 1:** Baseline Characteristics of the Study Population According to PAD Status Based on ABI Assessment

	NoPAD (n = 15,676)	PAD (Standard) (n = 3,150)	PAD (Alternative) (n = 3,048)
Group definition	ABI-HIGH >0.9	ABI-HIGH ≤0.9	ABI-HIGH >0.9
	and ABI-LOW >0.9	and ABI-LOW ≤0.9	and ABI-LOW ≤0.9
Characteristic			
Male [*n* (%)]	6377 (40.7)	1549 (49.2)	1228 (40.3)
Age (years)	60.5 ± 8.9	62.8 ± 8.5	61.4 ± 8.5
Myocardial infarction [*n* (%)]	1426 (10.4)	593 (21.0)	386 (13.5)
Stroke [*n* (%)]	682 (5.0)	214 (7.7)	160 (5.7)
Peripheral vascular disease [*n* (%)]	766 (5.6)	934 (32.8)	319 (11.2)
Blood glucose (mmol/l)	6.19 ± 2.2	6.6 ± 2.6	6.2 ± 2.2
Diabetes mellitus [*n* (%)]	4719 (30.1)	1271 (40.4)	976 (32.0)
Serum cholesterol (mmol/l)	5.3 ± 1.2	5.4 ± 1.3	5.3 ± 1.2
Hyperlipidemia [*n* (%)]^b^	8144 (43.5)	1696 (53.8)	1396 (45.8)
GFR (ml/min)	75.2 ± 15.6	72.5 ± 17.7	74.1 ± 16.3
GFR <60 ml/min [*n* (%)]	2893 (15.4)	652 (20.7)	529 (17.4)
Serum uric acid (μmol/l)^b^	310 ± 90	325 ± 101	312 ± 95
Hyperuricemia [*n* (%)]^c^	3352 (17.9)	471 (14.5)	416 (13.5)
Smoking [*n* (%)]	2864 (18.0)	1000 (31.8)	685 (21.5)
BMI (kg/m^2^)	28.0 ± 5.0	28.9 ± 5.3	28.9 ± 5.2
Obesity based on BMI [*n* (%)]^d^	8319 (44.4)	1293 (41.0)	1240 (40.7)
SBP (mmHg)	138.7 ± 19.5	143.8 ± 21.5	140.7 ± 20.7
DBP (mmHg)	82.6 ± 9.7	83.1 ± 10.4	83.5 ± 10.1
Number of antihypertensives	1.69 ± 1.36	2.00 ± 1.40	1.71 ± 1.40
BP control [*n* (%)]^e^	8377 (44.7)	1073 (34.0)	1266 (41.5)

Data are presented as *n* (%) or mean ± SD unless otherwise indicated.

ABI, ankle–brachial index; GFR, glomerular filtration rate.

^a^Defined as fasting plasma glucose level ≥7 mmol/l, or known previous condition, or concomitant antiglicemic medication.

b Defined as serum cholesterol ≥ 6.5 mmol/l, or known previous condition, or concomitant antilipemic medication.

c Defined as serum uric acid level at or above the cutoff (363 μmol/l female individuals, 488 μmol/l male individuals), or known previous condition, or concomitant antiuricosuric medication).

d Defined as BMI ≥ 30 kg/m^2^.

e Definition based on European Society of Hypertension Guidelines (BP<140/90 mmHg).

**FIGURE 2:**
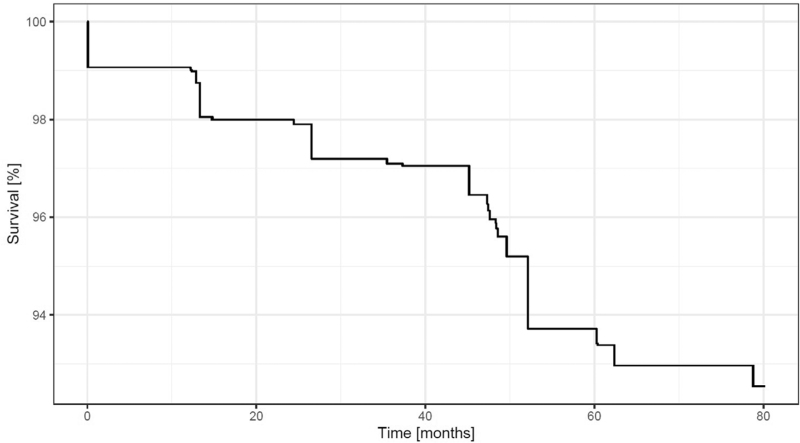
Overall survival of patients.

Of the patients, 1190 (5.4%, 12.7 death per 1000 patient-years) died during the follow-up period (median 1823 days, QR: 1786–1845). Death rates were 10.1, 25.7, and 13.6 per 1000 patient-years in patients without PAD, patients with PAD based on the traditional ABI assessment, patients with PAD based on the alternative ABI calculation exclusively, respectively. The survival curve is shown in Fig. 2.

**FIGURE 3:**
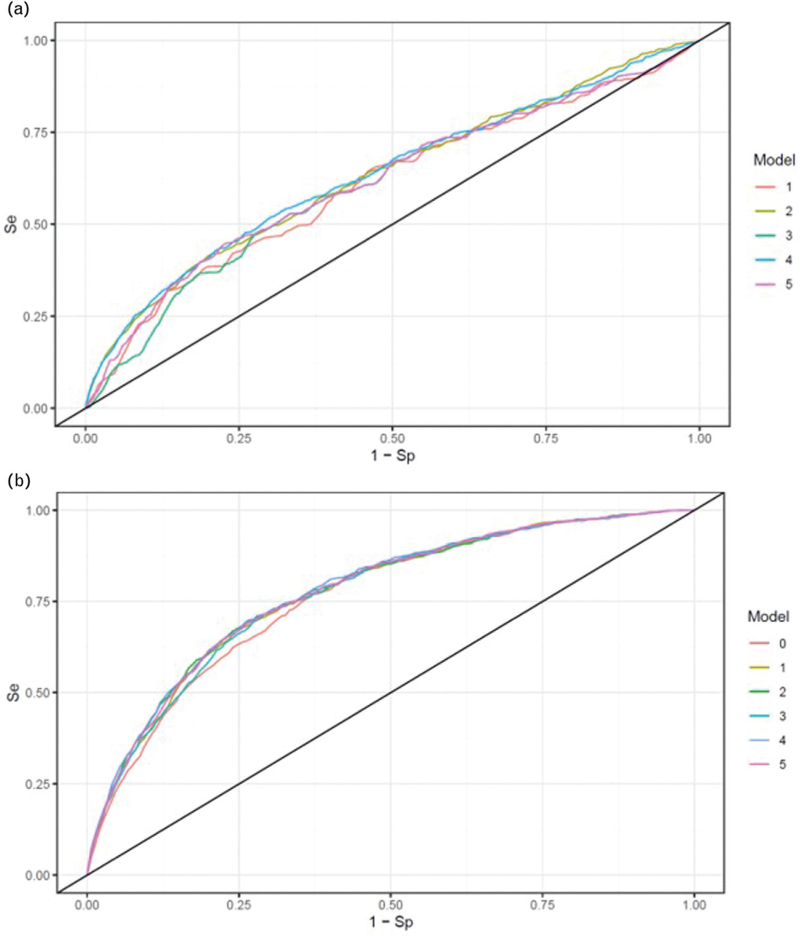
Receiver-operating curves of mortality prediction models. (a) Based on only ABI information (models 1–5). (b) based on ABI information (models 1–5) complemented by demographic and clinical parameters. Model 0 included only these latter parameters.

First, we evaluated the predictive performance of different ABI assessment methods alone in mortality prediction. The models were: ABI-HIGH ≤0.9 (binary variable); ABI-HIGH as a continuous variable; ABI-LOW ≤0.9 (binary variable); ABI-LOW as a continuous variable; and the number of affected ankle vessels (1–4). Time-dependent ROC curves at 3 years are shown in Fig. 3a. The corresponding AUCs were 0.609, 0.633, 0.608, 0.635, and 0.620, respectively.

Second, to simulate a realistic clinical scenario, we assessed the added predictive value of these ABI indices when incorporated alongside established clinical predictors of mortality in hypertensive patients (age, SBP and DBP, BMI, sex, diabetes, renal failure, prior myocardial infarction, and stroke). The clinical model without any PAD assessment was labeled Model 0. Time-dependent ROC curves for these combined models at 3 years are depicted in Fig. 3b, with corresponding AUCs of 0.763 (model 0), 0.774 (model 1), 0.777 (model 2), 0.772 (model 3), 0.780 (model 4), and 0.776 (model 5).

The predictive performance of the individual explanatory variables in the adjusted Cox proportional hazards models is summarized in Table 2 for categorical variables. The effects of ABI-HIGH and ABI-LOW as continuous variables on mortality risk are illustrated in Supplemental Figure 3.

**TABLE 2:** Hazard ratio (with 95% confidence interval) of mortality determined by predictive variables with categorical or ordinal values

	Model 0	Model 1	Model 2	Model 3	Model 4	Model 5
Male	2.18 (1.9–2.5)^*^	2.12 (1.8–2.5)^*^	2.1 (1.77–2.42)^*^	2.15 (1.83–2.53)^*^	2.07 (1.75–2.45)^*^	2.12 (1.78–2.5)^*^
Diabetes^a^	1.49 (1.31–1.69)^*^	1.43 (1.25–1.65)^*^	1.42 (1.24–1.63)^*^	1.45 (1.27–1.66)^*^	1.41 (1.23–1.63)^*^	1.44 (1.25–1.65)^*^
Renal failure^b^	1.95 (1.68–2.49)^*^	1.92 (1.63–2.27)^*^	1.92 (1.63–2.26)^*^	1.92 (1.64–2.26)^*^	1.89 (1.6–2.24)^*^	1.9 (1.62–2.25)^*^
Previous stroke	1.03 (0.8–1.33	0.97 (0.75–1.27)	0.95 (0.73–1.23)	0.99 (0.77–1.29)	0.95 (0.73–1.24)	0.95 (0.75–1.1.26)
Previous AMI	1.39 (1.18–1.64)^*^	1.32 (1.12–1.56)^*^	1.3 (1.11–1.53)^*^	1.32 (1.29–1.56)^*^	1.3 (1.1–1.52)^*^	1.3 (1.1–1.54)^*^
PAD based on ABI-HIGH		1.87 (1.59–2.2)^*^				
PAD based on ABI-LOW				1.6 (1.39–1.84)^*^		
1 vessel						1.16 (0.93–1.45)^*^
2 vessel						1.65 (1.33–2.0)^*^
3 vessel						1.72 (1.31–2.25)^*^
4 vessel						2.1 (1.69–2.59)^*^

ABI, ankle–brachial index; AMI, acute myocardial infarction; PAD, peripheral artery disease.

a Diabetes defined by fasting plasma glucose ≥7 mmol/l or known diabetes or antiglycemic medication.

b Renal failure defined as glomerular filtration rate <60 ml/min.

* Statistically significant, *P* < 0.05.

Using the adequacy index, we quantified the added predictive information contributed by the different ABI assessment methods for mortality risk. This incremental value ranged from 6 to 11%, indicating a modest additive benefit. Among the ABI metrics, ABI-LOW as a continuous variable demonstrated the greatest value.

Furthermore, we conducted a supplementary analysis excluding all patients identified with PAD by the traditional ABI-HIGH method, resulting in a cohort of 16 341 individuals. Within this subgroup, the predictive performance of the alternative ABI assessments (ABI-LOW and vessel-specific ABI) was evaluated. The AUCs, based on models without other clinical covariates, were 0.545 for ABI-LOW as a binary variable, 0.56 for ABI-LOW as a continuous variable, and 0.545 for the vessel-specific ABI (number of affected ankle vessels). These AUCs suggest limited, likely negligible predictive value, consistent with the modest additive value observed in the overall analyses.

A further subgroup analysis was conducted, restricting the sample to patients with diabetes (6405 participants). Within this subgroup, AUCs of models without clinical covariates were 0.587 (model 1), 0.626 (model 2), 0.604 (model 3), 0.639 (model 4), and 0.608 (model 5). These AUCs reveal that predictive performance in this subgroup was very similar to that in the overall cohort.

## DISCUSSION

Building on our prior reports of PAD prevalence [11] and the prognostic value of the ABI in a large-scale hypertensive cohort [12], this study, first in the literature, evaluated the prognostic performance of alternative ABI calculation methods for mortality risk stratification in hypertensives.

In nearly 22 000 hypertensive individuals from the ÉRV program, ABI based on the lower ankle pressure (ABI-LOW) identified twice as many PAD cases (28.3 vs. 14.4%) compared with the standard higher ankle pressure method (ABI-HIGH), consistent with findings from other clinical and population-based studies [16–18,21,28,29]. This raises the possibility of substantial underdiagnosis with the standard approach. A multivessel ABI assessment – based on the number of arteries (0–4) with abnormal readings – further improved characterization of lower extremity atherosclerotic burden [20]. Thus, alternative approaches – including ABI-LOW, use of ABI as a continuous variable, and multivessel ABI assessment – may enhance the prognostic utility of ABI in mortality prediction.

Prior to addressing our results in this regard, it is essential to clarify two key issues that may considerably influence their interpretation: first, the assessment of the mortality risk level in our study sample, and second, the choice of study endpoint (all-cause mortality) that may strongly influence both the interpretation and generalizability of the results.

An important consideration is the extent to which the mortality data revealed in our study characterize the investigated population as bearing a high or rather a low-risk profile. International evidence indicates a broad range of all-cause mortality rates among hypertensive populations (14.3–18.1 per 1000 person-years in the United States NHANES cohorts [30], 22.4 per 1000 person-years in a cohort of 3.5 million newly diagnosed hypertensive [31] and 7.17 per 1000 person-years in stage I hypertensive [32]). Notably, variations in survival across studies largely reflect differences in demographic characteristics, comorbidity patterns, and prevailing blood pressure levels [33].

Although our subgroup cohort with PAD (based on standard diagnosis) showed a mortality rate (25.7 per 1000 patient-years) that may seem unexpectedly high, prior evidence indicates that mortality among patients with PAD can be even greater. Notably, in a systematic review of 124 randomized and observational studies, summarizing outcomes of patients with PAD (ABI < 0.9; mean age 68 years), authors reported an all-cause mortality rate of 81 (39–173) per 1000 patient-years [34]. It is plausible that PAD cases identified in our cohort predominantly represented less severe disease (asymptomatic or mildly symptomatic). Nevertheless, it is also well established that the prognosis of asymptomatic PAD is comparable to that of patients with intermittent claudication [35]. In light of these findings, our study population appears to have a relatively moderate risk profile.

Regarding the study endpoint (all-cause mortality), the absence of cardiovascular-specific mortality data limits interpretability and generalizability. Cause-specific mortality was not available in our dataset. However, the literature generally views all-cause and cardiovascular mortality as complementary rather than hierarchical endpoints. All-cause mortality is a robust measure with minimal misclassification risk but may dilute cardiovascular effects due to non-cardiovascular deaths, requiring longer follow-up. In contrast, cardiovascular mortality offers greater specificity and statistical power when interventions target cardiovascular outcomes, and it fits well in composite endpoints. Still, it is prone to misclassification (e.g. death certificates, ICD coding), potentially introducing bias and overstating net benefit by overlooking noncardiovascular harms [36,37].

Taking these considerations into account, our study found that, in adjusted survival models for all-cause mortality prediction, both ABI-HIGH-defined and ABI-LOW-defined PAD were independently associated with the risk of death, regardless of whether they were evaluated as dichotomous or continuous variables.

Furthermore, the number of vessels involved, quantified by the multivessel ABI score (ranging from 0 to 4), exhibited a clear dose–response relationship with mortality risk. Individuals with involvement of all four arteries faced a two-fold increase in mortality hazard, comparable to that associated with renal dysfunction, a well established predictor of mortality. These findings are consistent with prior results from the Multi-Ethnic Study of Atherosclerosis (MESA), [20] reinforcing the notion that the extent of arterial involvement serves as a robust marker of systemic atherosclerotic risk.

Despite these associations, the overall discriminatory power of ABI measures alone was modest. AUC values ranged from 0.608 to 0.635, highlighting the limited predictive capacity of ABI in isolation. When combined with traditional risk factors, AUCs improved to 0.763–0.780, indicating meaningful but not exceptional gains. Similarly, ABI indices added only slight prognostic value (6–11%) beyond conventional markers, consistent with prior studies reporting similar AUCs and incremental improvements using ABI-LOW or multivessel ABI [18,21,28].

A key clinical question is whether ABI-LOW offers added prognostic value over ABI-HIGH for mortality risk assessment. Although ABI-LOW classified more patients as having PAD, its AUCs and overall model performance were not significantly different from ABI-HIGH – even in subgroup analyses excluding traditionally defined PAD or focusing on diabetic patients, who may have distal atherosclerosis. Notably, individuals identified as PAD-positive only by ABI-LOW had mortality rates comparable to PAD-negative patients, indicating limited prognostic discrimination. Prior studies likewise report no consistent advantage of ABI-LOW for all-cause mortality prediction [16,18,21,28], though some evidence suggests a potential benefit for cardiovascular mortality [21].

Current hypertension guidelines [1,2] emphasize comprehensive cardiovascular risk assessment, particularly the refinement of risk stratification through evaluation of HMODs, but offer little direction on how different vascular damage assessments should be selected, sequenced, or combined in practice.

In this context, our findings raise the question of whether alternative ABI calculations – representing different interpretations of standard Doppler measurements – may offer clinical utility. While we observed no improvement in mortality prediction, such approaches might help identify subgroups warranting closer monitoring or further vascular evaluation. Emerging evidence [6,7,38] indicates that combining ABI with pulse wave velocity (PWV) enhances cardiovascular risk stratification. Thus, integrating ABI with vascular stiffness indices in selected hypertensive populations, potentially identified via alternative ABI metrics, may provide additional prognostic value and merits further study.

### Limitations

Our findings must be interpreted in light of certain limitations. While ABI measurements were standardized and preceded by structured training at all study sites, potential misclassification bias cannot be excluded in a study of this scale. Follow-up was incomplete in approximately 20% of participants, though appropriate interval-censored modeling was used to mitigate bias. The relatively short follow-up period (median ~5 years) and the relatively low-risk profile, low number of death events in the cohort may have attenuated our ability to detect stronger prognostic signals. Longer term follow-up and external validation in more diverse hypertensive populations are warranted. Finally, the fact that the outcome was restricted to all-cause mortality, without consideration of cardiovascular mortality or other hard endpoints, also represents a limitation in the interpretation and generalizability of our study.

### Future directions

Future prospective studies should assess the role of ABI-LOW and multivessel scoring in predicting cardiovascular-specific outcomes, ideally with longer term follow-up, higher risk status, and integration of other vascular damage indices.

In conclusion, in this large cohort of hypertensive patients, alternative ABI methods – ABI-LOW and multivessel scoring – identified a greater number of PAD cases and were independently associated with all-cause mortality. However, consistent with findings from other populations, and now analyzed specifically in hypertensive patients, their added predictive value over the standard ABI-HIGH approach was modest, offering no significant improvement in mortality risk discrimination. These results suggest that while ABI-LOW may have limited utility for all-cause mortality prediction, it could help identify higher risk subgroups who may benefit from further vascular assessment.

## ACKNOWLEDGEMENTS

This manuscript is original, has not been published previously, and is not under consideration by any other journal. All authors have read and approved the final version.

Source of funding: This work was supported by the EGIS Pharmaceuticals PLC.

### Conflicts of interest

There are no conflicts of interest.

## Supplementary Material

Supplemental Digital Content
