# Supplementary material for: Alternative ankle–brachial assessments show no significant added value in predicting mortality of hypertensive patients
Source: J Hypertens. 2026 Feb 9;44(4):682–90. doi: 10.1097/HJH.0000000000004255 (PMC12955972; doi:10.1097/HJH.0000000000004255)
Supplement: Supplemental Digital Content [file jhype-44-682-s001.docx]

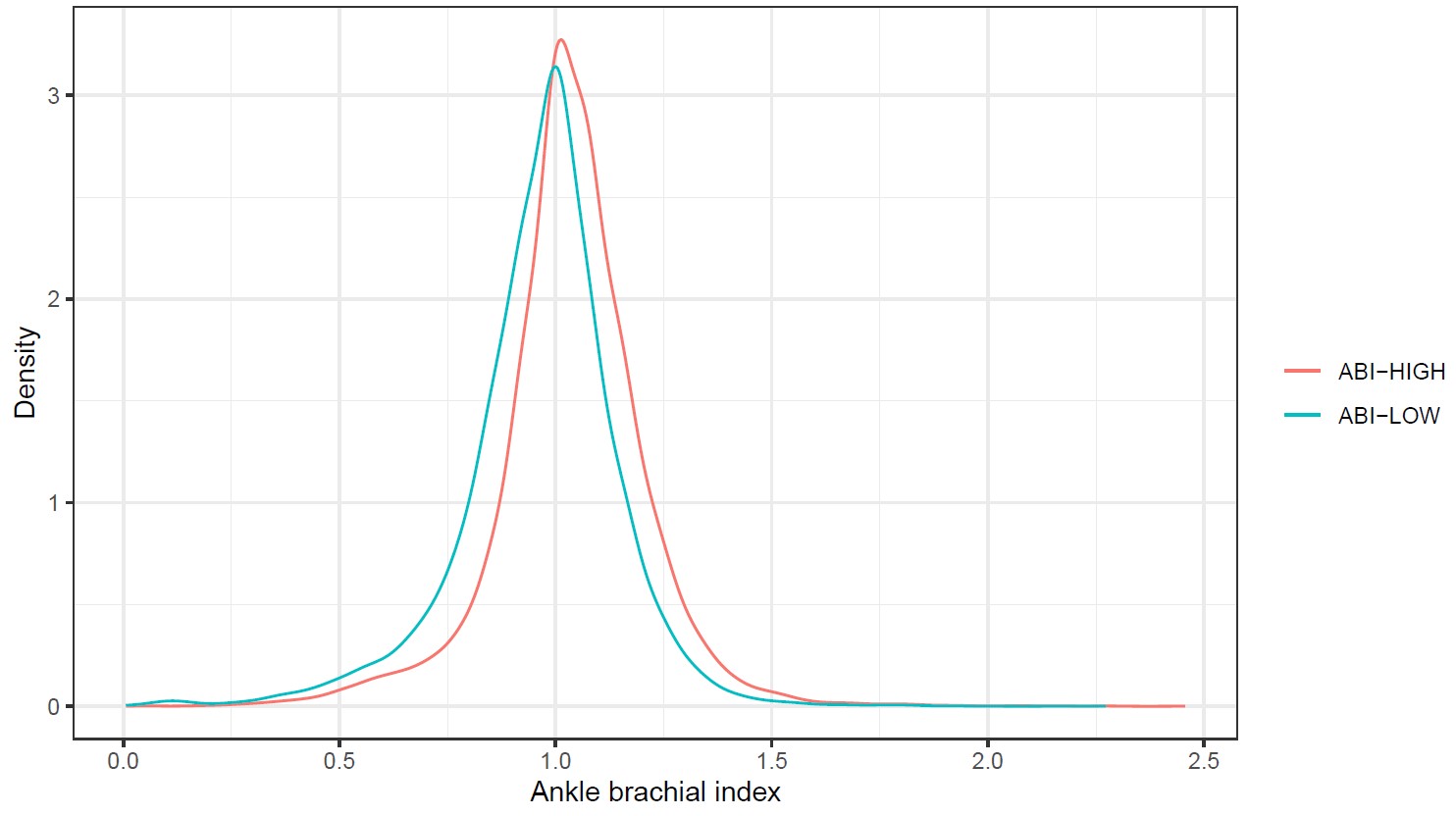


**Supplementary Figure 1. Density distribution of ABI values (traditional-ABI-HIGH, alternative-ABI-LOW**


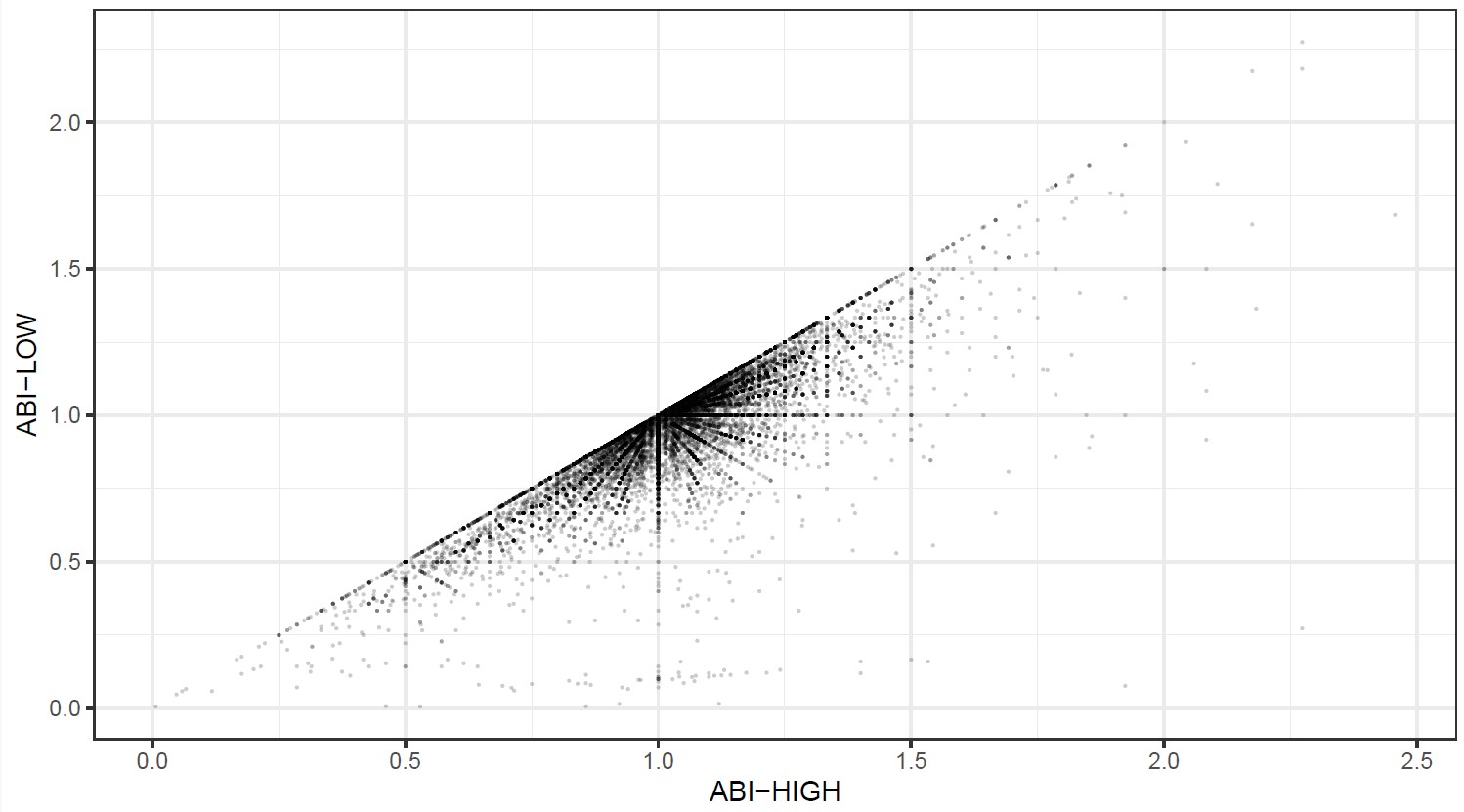


**Supplementary. Figure 2. Relationship between the ankle brachial index (ABI) measured using the higher ankle pressure (ABI-HIGH) vs. the lower ankle pressure (ABI-LOW)**


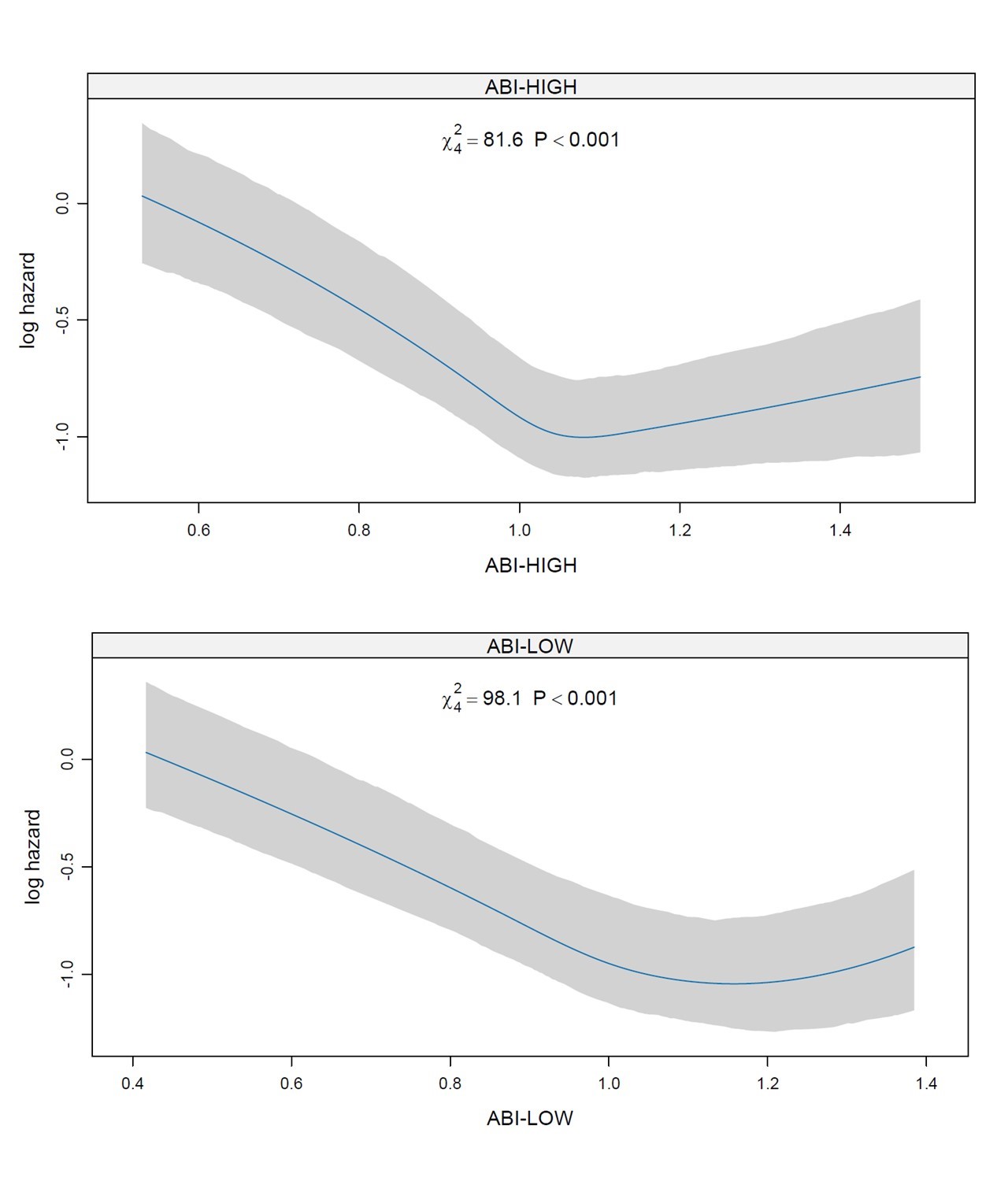


**SupplFigure3. The hazard of mortalitydeterminedby ABI-HIGH and ABI-LOW ascontinuousvariables (Models 2 and 4)**
